# Trunk angular velocity: A convenient, valid and responsive substitute for force plate-based measures of dynamic postural stability

**DOI:** 10.1371/journal.pone.0323993

**Published:** 2025-05-27

**Authors:** Lammert A. Vos, Maarten R. Prins, Elien Plompen, Jaap H. van Dieën, Idsart Kingma

**Affiliations:** 1 Research and Development, Military Rehabilitation Center ‘Aardenburg’, Doorn, The Netherlands; 2 Department of Human Movement Sciences, Faculty of Behavioural and Movement Sciences, Vrije Universiteit Amsterdam and Amsterdam Movement Sciences, Amsterdam, The Netherlands; Iran University of Medical Sciences, ISLAMIC REPUBLIC OF IRAN

## Abstract

**Objective:**

Deficits in dynamic postural stability are associated with increased risk of lower limb injuries. Objective dynamic postural stability assessments typically require force plates, which are not commonly available in the clinic. Moreover, these outcomes are not directly related to balance strategies, which hampers clinical interpretation and translation to therapeutic goals. Direct measurements of kinematics can be more suitable in these respects. This study aims to investigate the concurrent validity and responsiveness of trunk angular velocity.

**Methods:**

Data from fourteen healthy participants were analyzed using a within-subject design. Participants were standing on one leg on a platform with an embedded force plate that was used to impose mediolateral perturbations to evoke balance responses. To introduce variability within participants, perturbation amplitude and base-of-support width were manipulated. Time-to-stability, mean center of pressure speed and the mean vertical force deviation of the ground reaction force were derived from force plate data and mean trunk angular velocity was obtained from an inertial measurement unit. All outcomes were calculated over a five-second time window after perturbation onset. Multilevel correlations were calculated to assess the concurrent validity of trunk angular velocity. To compare the responsiveness of trunk angular velocity and force plate-based outcomes, effect sizes of a repeated measures ANOVA were used.

**Results:**

Trunk angular velocity correlated strongly with center of pressure speed (r = 0.78) and the vertical force deviation (r = 0.74) and moderately with time-to-stability (r = 0.61). Trunk angular velocity demonstrated the largest effect sizes in the repeated measures ANOVA.

**Conclusion:**

Trunk angular velocity is a promising outcome to use in the clinic. Its concurrent validity and responsiveness are supported by the results of this study.

## 1. Introduction

Deficits in dynamic postural stability are associated with increased risk of lower limb injuries [1–3]. Conversely, lower limb injuries can affect dynamic postural stability [4], with a high likelihood of re-injury [5–8] and increases the risk of other lower limb injuries [9]. Postural stability tests can help to pinpoint those individuals that have the highest risk of sustaining (re-)injury [1].

This study utilizes mediolateral platform perturbations, imposed by a movable platform, to study dynamic postural stability [10–12] whilst standing on one leg. Multiple mediolateral platform perturbations with randomly distributed time intervals are used to evoke and measure balance responses over a five-second time window after each perturbation using an embedded force plate. The point of application (center of pressure: CoP) and the magnitude of the ground reaction force (GRF) are used to calculate various reliable outcome measures [11].

The aforementioned postural stability outcomes require a force plate [10–12] which is common practice in studies using postural stability tests [1,13–16]. However, force plates are usually unavailable in the clinic and force plate-based outcomes are not directly related to balance strategies [17], which hampers clinical interpretation and translation to therapeutic goals. There are two mechanisms to restore balance whilst standing on one leg: the ‘move the CoP’ mechanism, in which the CoP is moved with respect to the center of mass (CoM) while the angular momentum is preserved and the ‘counter-rotation’ mechanism, in which the trunk, legs and arms are used as a counter movement, changing the angular momentum around the CoM [18]. In the clinic, therapists generally rely on visual observations to assess performance during postural stability tasks. Trunk movements can be considered a suitable target for observation, because they are relatively large and slow and are related to the counter-rotation mechanism [18]. Moreover, it has been found that the measurement of trunk movements during stance is a reliable and simple way to assess balance control [19]. In general, increased trunk movement is an indication of a reduced single leg stability [18] and increased trunk movement during a drop jump is associated with an increased risk of knee injuries [20]. Furthermore, a focus on trunk movements, for example using inertial measurement units [21,22], might even allow for field assessment of dynamic postural stability during sports. However, it is currently unclear how trunk movements are related to force plate-based postural stability outcomes. To the best of our knowledge, this relationship has not been studied to date.

This study aimed to assess the concurrent validity of trunk angular velocity (TAV) in measuring dynamic postural stability and its responsiveness to varying levels of difficulty. The correlation between several reliable force plate-based outcomes and TAV was calculated to determine the concurrent validity. Changes in the CoP speed (CoPS) and the magnitude of the GRF reflect movements of the whole body, to which trunk motion likely contributes a significant amount. Therefore, our hypothesis was that TAV is strongly correlated to force plate-based outcomes. Additionally, responsiveness of TAV to variations of the amplitude of the imposed perturbations and to changes of the width of the base of support (BoS) was compared to force plate-based outcomes. We hypothesized that responsiveness of TAV to these variations is at least equal to the responsiveness of force plate-based outcomes.

## 2. Methods

### 2.1 Participants

Eighteen healthy participants were included in this study. Participants were recruited at the VU University in Amsterdam, the Netherlands. Nine males and nine females, with a mean age of 21.9 (19–31) years, mass of 73.5 (51–108) kg, who participated in sports 4.3 (2–9) times a week, were included. Exclusion criteria were: injury of the lower limb within six months prior to this study, anterior cruciate ligament injury in the past, a disorder or medication use that could affect postural control, or any other condition that rendered a participant unfit to be tested.

The study was approved by the Scientific and Ethical Review board of the Faculty of Behavioral and Human Movement Sciences of the VU University in Amsterdam, the Netherlands (VCWE-2020-052). All measurements took place between January 2022 and May 2022. All participants provided written informed consent before measurements took place.

### 2.2 Equipment

The test was performed on the Dynamic Stability and Balance Learning Environment (DynSTABLE, Motek Medical, the Netherlands), which consists of a moveable platform of 1 by 1 meter with an integrated uniaxial force plate. A screen in front of the platform was used to provide information regarding switching legs and remaining resting time during the test.

TAV was measured using an Xsens motion capture sensor (MVN Awinda, Movella, the Netherlands) attached to the back of the participant at approximately the level of vertebra T11 using a Velcro strap. The force plate data were synchronized with the platform perturbations by the manufacturer’s control and data acquisition software (D-Flow, Motek Medical, the Netherlands). Xsens data were recorded separately. Therefore, an additional Xsens sensor was placed on the platform to detect the platform perturbations through the resulting acceleration signal. The two Xsens sensors were synchronized during recording using the manufacturers (MTw Awinda, Movella, the Netherlands) software. The platform sensor was used to synchronize the Xsens data with the force plate data.

### 2.3 Procedure

Participants were tested on bare feet. Socks were worn during the trials with wooden footboards. For safety reasons, each participant wore a safety harness that provided no weight support unless the participant would fall.

**Fig 1:**
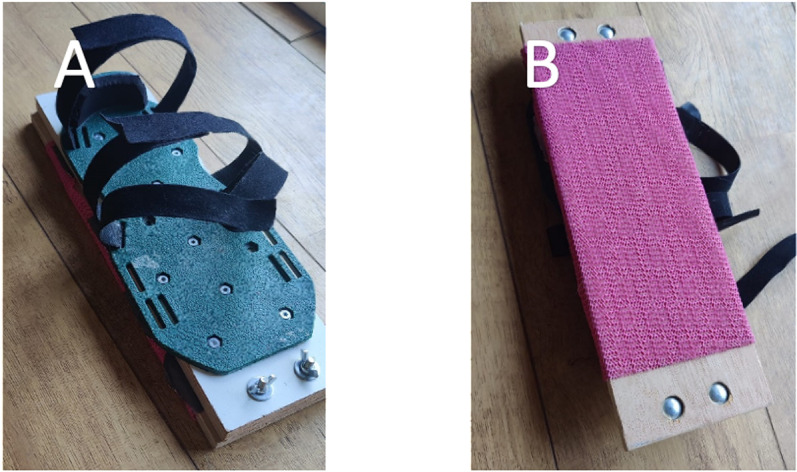
Wooden footboards to alter the width of the base of support during the test. A: Top of the board, which was attached to the foot with Velcro straps. B: Bottom of the board, which could be removed and replaced by a board with a different width. An anti-slip mat was attached at the bottom of the board.

Mediolateral perturbations with a magnitude of 2, 3.5 and 5 cm with a maximum speed of 0.127, 0.216 and 0.308 m/s respectively were imposed and the width of the BoS was varied by strapping wooden footboards of 6, 9 and 12 cm wide under the stance foot during the test (Fig 1).

**Fig 2:**
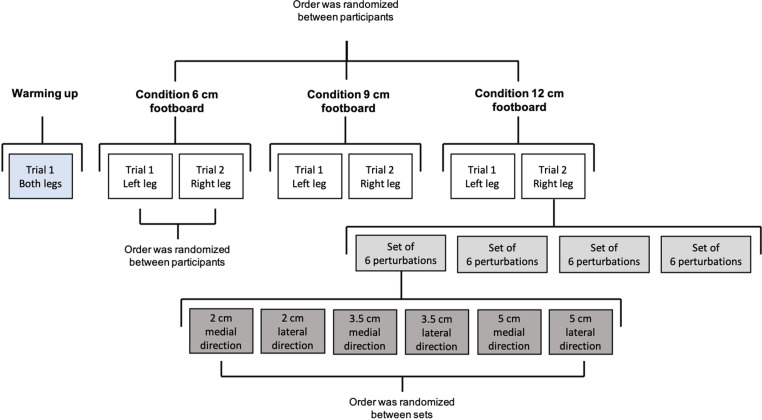
Sequence of the conditions during the experiment.

The experiment consisted of a warming up trial, intended to reduce learning effects during the experiment [12], and 3 testing conditions, one for each of the footboards. Each of the testing conditions consisted of two trials: one on the right leg and one on the left leg (Fig 2). Participants with an even identification number began on the right leg, and participants with an odd number began on the left leg. The order of the footboards was randomized across participants.

The warming up trial consisted of a total of 24 perturbations, divided into four sets of six perturbations varying in magnitude (2 cm, 3.5 cm and 5 cm) and direction (medial and lateral). The magnitudes and directions were applied in randomized order. Participants were standing on one leg without a foot board during the warming up trial. A twenty second rest was imposed after each set, during which the participant stood on two legs. After the rest, the participant switched legs. Each of the six experimental trials (two legs x three footboards) consisted of the same sets of perturbations as the warming up trial, however, all four sets within one trial were performed on the same standing leg. Each condition consisted of two trials (one for each leg), resulting in 48 perturbations per condition. The time between perturbations within a set was quasi-randomized between 5.5 and 7.5 s in such a way that each trial lasted four minutes). Participants were not informed in advance about the different magnitudes of the perturbations and did not know the order of the footboards in advance.

During the trials, information about the test was projected on a screen in front of the platform. Information was provided about when to switch legs and what the remaining resting time was. Participants were asked to keep their swinging leg off the ground and to avoid pressing the swinging leg against the stance leg. The hand/arms position was not constrained. A point was projected on the screen at approximately eye level, which participants could use as a point of focus during the trials. Participants were asked not to look down and to keep their eyes open. Focusing on the point on the screen was not mandatory.

Participants were asked to regain their balance on one leg as quickly as possible following the perturbations. If necessary, participants were allowed to place both feet on the ground or use the harness to hang in. In these cases, the responses were recorded as imbalanced. Balance responses were also considered imbalanced if the stance foot was shifted or if a hop was used. Imbalances were recorded manually.

### 2.4 Outcome measures

Mean trunk angular velocity (TAV) was assessed with an inertial measurement unit and time-to-stability (TTS), mean center of pressure speed (CoPS) and the mean force variation of the vertical component of the ground reaction force (Force Y deviation: FYdev) were assessed with a force plate. All outcomes were calculated over a five-second time window after each perturbation.

- TAV is the mean total angular velocity, i.e. the mean Euclidian length of the angular velocity vector of the trunk.
- CoPS is the average speed of the center of pressure. CoPS is calculated as the total path length divided by measurement time of CoP.
- TTS is the time for a subject to return to a stable state after a perturbation. TTS is defined as the last instance that the vertical component of the GRF enters a predetermined range (+/- 2.5% of bodyweight) and stays within that range for one full second, with a maximum possible score of 4 seconds. TTS is based on the magnitude of the GRF.
- FYdev: the mean variation of the vertical component of the GRF, i.e., the mean of the absolute vertical force minus body weight.

### 2.5 Data analysis

Force plate data were collected at a rate of 1000 samples/s. Data recording started 0.2 s after the initiation of each perturbation up to 5.2 s after perturbation onset. Raw force data were filtered offline with a second-order unidirectional low-pass Butterworth filter with a cut-off frequency of 12 Hz [11,12]. Average CoPS was calculated by dividing the total length of the CoP trajectory by the measurement time window. TTS was calculated as the time elapsed from perturbation until the vertical component of the GRF remained within a 2.5% margin of the body weight for 1 s [11]. FYdev was calculated as the mean value of the absolute difference between the vertical component of the GRF and body mass and expressed as percentage of body mass over the measurement time window after a perturbation.

Xsens data were collected at a rate of 100 samples/s. Data recording started 0.2 s after the initiation of each perturbation up to 5.2 s after perturbation onset. The data were interpolated for missing samples and filtered with the same filter as the force plate data. Total angular velocity of the trunk was calculated from the three-dimensional measured angular velocities using the Pythagorean Theorem.

Perturbations after which a participant was imbalanced were discarded from all analyses. If more than 80% of the perturbations over the entire experiment were imbalanced, all data of that participant were discarded.

### 2.6 Statistical analysis

Correlation coefficients were calculated for the associations of all six possible combinations of the four outcome measures (TAV, CoPS, TTS and FYdev) with a multilevel correlation [23]. Multilevel correlations were used to account for differences between conditions (perturbation amplitude and BoS width). Multilevel correlations were calculated using all datapoints of each participant (144 perturbations x 18 participants), i.e., one per outcome per perturbation, using a custom RStudio package [24]. In addition to the multilevel correlation, within participants correlations were calculated and shown in a figure to compare individual correlations with the multilevel correlations, to gain insights in the variance between participants. Within participants correlations were calculated using all individual datapoints of each participant (6 trials x 24 perturbations). All within participant correlation coefficients were transformed using Fisher’s Z transformation to calculate average participants correlations. The mean value was calculated over transformed values. Thereafter, the mean values were back-transformed to obtain the average correlation coefficients. To interpret the correlations, r = 0.00 – 0.10 were regarded as negligible, r = 0.10 – 0.39 as weak, r = 0.40 – 0.69 as moderate, r = 0.70 – 0.89 as strong and r = 0.90 – 1.00 as very strong [25].

A 3x3 repeated measures ANOVA for each of the four outcome measures was used to determine if perturbation amplitudes and/or BoS widths contributed to variation in scores. Partial Eta Squared (η^2^_p_) was calculated as a measure of effect size and thereby responsiveness of the outcome measures. To interpret the effect sizes, η^2^_p _ = 0.01 were regarded as small, η^2^_p _ = 0.06 as medium and η^2^_p _ = 0.14 as large [26].

The alpha level was set at 0.05. Data analysis was performed using MATLAB (the Mathworks, Natick, USA), multilevel correlations were calculated using RStudio (Vienna, Austria), ANOVAs were performed using Jamovi (Sydney, Australia).

## 3. Results

One male participant was excluded from data analysis due to problems with equipment. Three female participants were imbalanced in more than 80% of the perturbations and therefore excluded from further analyses. Hence, fourteen participants were included in the data analysis. The average imbalance rate for lateral perturbations was 49.4%, ranging from 13.7%–84.8% for the smallest to largest perturbation amplitude respectively. For medial perturbations the average imbalance rate was 10.0%, ranging from 1.79%–23.2%. Because of the high imbalance rates for lateral perturbations, statistical analyses were only performed for medial perturbations, resulting in a total of 72 datapoints (6 trials x 12 perturbations) per participant.

### 3.1 Correlations

**Fig 3:**
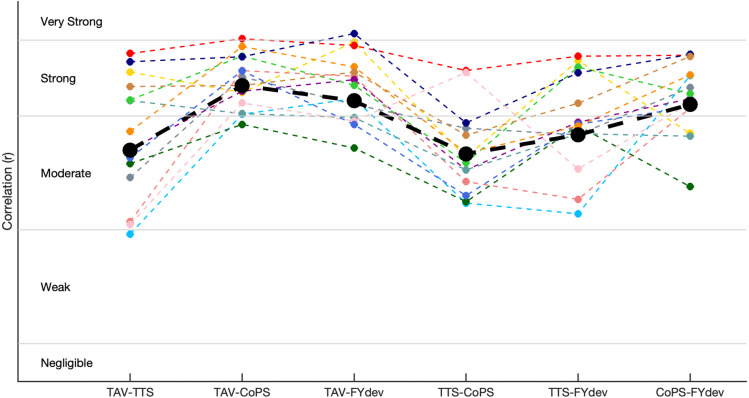
Correlation coefficients of multilevel correlations (black and bold) and within participants correlations (each color is one participant). Horizontal lines represent the interpretation of the correlations (negligible, weak, moderate, strong, very strong). CoPS = center of pressure speed, TAV = trunk angular velocity, FYdev = the mean variation of the vertical component of the ground reaction force, TTS = time-to-stability.

**Fig 4:**
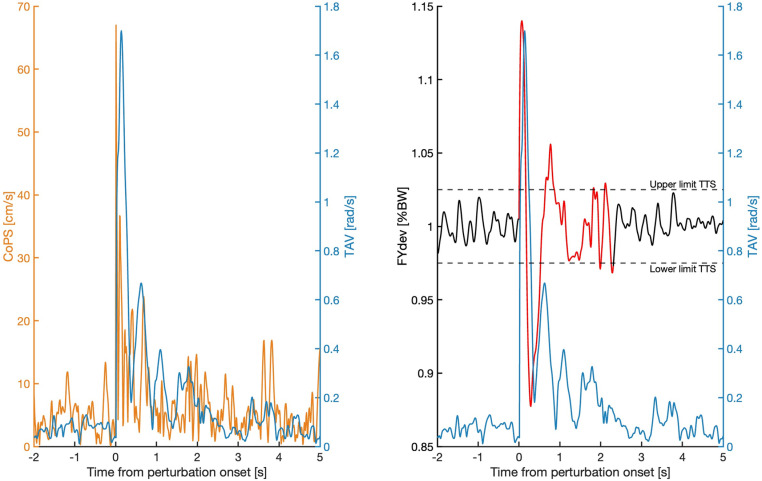
Typical example of the signals yielding the four outcome measures in the two seconds before and five seconds after a single perturbation. The FYdev is colored red from the perturbation onset until it first re-enters the threshold and remains within the threshold for at least one second. From that point onward, it is colored black. In this specific case the TAV score was 0.2 rad/s, the CoPS score was 7.1 cm/s, the FYdev score was 1.8 percentage of body weight and the TTS score was 2.3 s. CoPS = center of pressure speed, TAV = trunk angular velocity, FYdev = the mean variation of the vertical component of the ground reaction force, TTS = time-to-stability.

All multilevel correlations were moderate to strong (Table 1). TAV strongly correlated with CoPS (r = 0.78) and FYdev (r = 0.74) and moderately with TTS (r = 0.61). Individual within participant correlations followed a pattern similar to the multilevel correlations (Fig 3). Averaged over participants, within participant correlations were slightly higher than the multilevel correlations, ranging from 0.02 (TAV-COPS) higher to 0.06 (TAV-TTS) higher. Fig 4 shows a typical example of the three signals and four resulting outcome measures in the two seconds before and five seconds after a single perturbation.

**Table 1:** Multilevel correlations (r) and p-value of force plate-based outcomes and TAV. Strong correlations are bold. r = correlation coefficient, TAV = trunk angular velocity, TTS = time-to-stability, CoPS = center of pressure speed, FYdev = the mean variation of the vertical component of the ground reaction force.

	r (P-value)		
	TTS	CoPS	FYdev
CoPS	*0.60 (P* < *0.001)*		
FYdev	*0.65 (P* < *0.001)*	***0.73*** *(P* < *0.001)*	
TAV	*0.61 (P* < *0.001)*	***0.78*** *(P* < *0.001)*	**0.74** *(P* < *0.001)*

### 3.2 ANOVA

**Fig 5:**
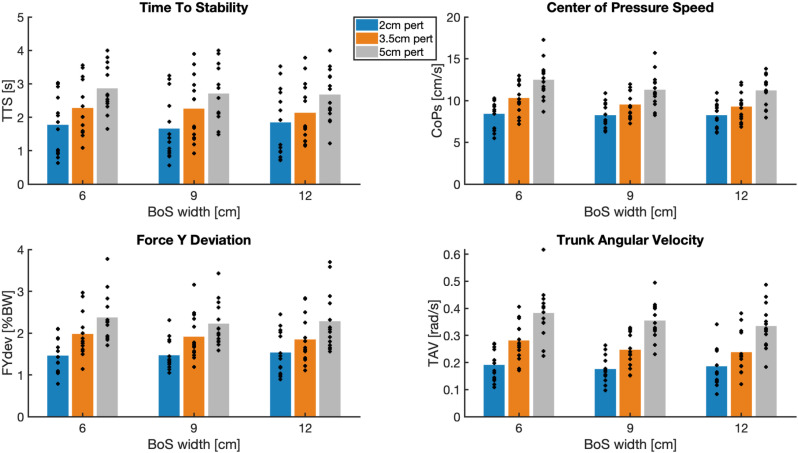
Bar graph with individual datapoints of force plate-based outcomes and TAV. Each dot represents the average outcome of one participant for the combination of base of support (BoS) width and perturbation amplitude (pert). Perturbations of 2, 3.5 and 5 cm are represented in blue, orange and grey respectively. CoPS = center of pressure speed, TAV = trunk angular velocity, FYdev = the mean variation of the vertical component of the ground reaction force, TTS = time-to-stability.

**Fig 6:**
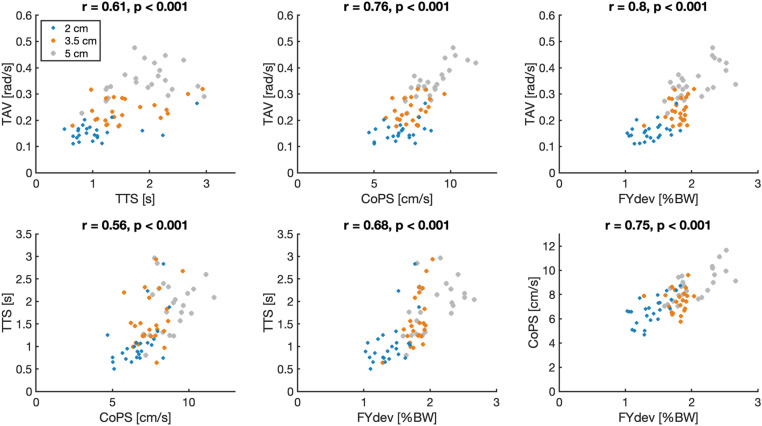
Scatterplots of force plate-based outcomes and TAV of a typical participant. Each dot represents one medial perturbation, resulting in 72 dots per scatterplot. Perturbations of 2, 3.5 and 5 cm are represented in blue, orange and grey respectively. CoPS = center of pressure speed, TAV = trunk angular velocity, FYdev = the mean variation of the vertical component of the ground reaction force, TTS = time-to-stability.

A significant effect of perturbation amplitude was found on TAV, CoPS, TTS and FYdev, as can be seen in Table 2 and Fig 5. The largest effect sizes of both perturbation amplitude and base of support were found for TAV. Compared to the smallest perturbation, the largest perturbation amplitude resulted in a 95.4% increase in TAV, a 41.1% increase in CoPS, a 57.7% increase in TTS and a 55.2% increase in FYdev. A small but significant effect of BoS width was found on TAV and CoPS (6% and 3.3% decrease in smallest compared to largest BoS, respectively), whilst no significant effect was found on TTS and FYdev. A significant interaction effect of perturbation amplitude and BoS width was found for CoPS. Fig 5 suggests that the effect of perturbation amplitude on CoPS was the largest for the smallest footboard. A typical example of the relation between TAV and force plate-based outcomes showed a substantial responsiveness to perturbation magnitude (Fig 6), similar among outcomes and a minor responsiveness to BoS width (S1 Fig).

**Table 2:** Results of the repeated measures ANOVA, comparing effect of perturbation amplitude and the width of footboards on force plate-based outcomes and TAV. Significance of the factors of the ANOVA are presented using P-values. Significant P-values are bold. Effect sizes of the factors of the ANOVA are presented using Partial Eta Squared values. Pert = perturbation, BoS = base of support.

Outcome measure	P-value (η^2^_p_)		
	Perturbation (Pert)	Base of Support (BoS)	Pert x BoS
CoPS	**<.001** (.878)	**.020** (.277)	**.036** (.189)
TTS	**<.001** (.794)	.506 (.055)	.324 (.091)
FYdev	**<.001** (.882)	.396 (.074)	.143 (.131)
TAV	**<.001** (.912)	**.003** (.392)	.085 (.154)

## 4. Discussion

The main aim of this study was to investigate the concurrent validity and responsiveness of TAV in response to single leg balance perturbations. Multilevel correlations between TAV and several force plate-based outcomes were calculated to investigate the concurrent validity. CoPS and FYdev showed a strong correlation with TAV and TTS showed a moderate correlation with TAV. Effect sizes of the repeated measures ANOVA were used to investigate the responsiveness to perturbation magnitude and width of the BoS. TAV showed large effect sizes for perturbation magnitude and BoS variation. TAV effect sizes were even larger than effect sizes of force plate-based outcomes, indicating that TAV is a responsive outcome for balance assessment.

The concurrent validity of TAV is supported by the results obtained in this study. The correlations with TAV for CoPS and FYdev were strong, demonstrating good agreement between force plate-based outcomes and TAV. The correlations between TTS and TAV were moderate. TTS is based on a binary criterium (FYdev within or outside the specified boundary). During the measurements, TTS appeared to be strongly influenced by small abrupt movements that often had no clear relationship with stabilization, such as briefly scratching the nose in the time-period after a perturbation. FYdev is a continuous measure and captured all movements in the timeframe after a perturbation. This may explain why TTS, despite the fact that it is based on the same signal as FYdev, had a much lower correlation with TAV than FYdev.

Within participant correlations were calculated to gain insights in the variance between participants. Within participant correlations deviated slightly from the multilevel correlation. However, the trendline, i.e., the individual correlation of all six combinations relative to each other, was for most participants comparable to the multilevel correlation trendline (Fig 3). However, some participants deviated from this trendline. Correlations with TTS had the largest variance between participants. This is possibly caused by the fact that some of the participants made small movements that had no clear relationship with stabilization, as described above. Not all participants made such movements. Consequently, the extent to which variation of TTS reflected postural stability varies over participants. The within participant correlations between CoPS and TAV resulted in the smallest variation over participants (ranging from r = 0.68 to r = 0.90).

Responsiveness of TAV was determined using effect sizes of the main effects of perturbation magnitude and BoS variation in the repeated measures ANOVA. Perturbation amplitude affected both TAV and the force plate-based outcomes. Larger perturbations resulted in higher values of these outcomes. Adjusting the BoS width only slightly but significantly affected TAV and CoPS, where a smaller BoS resulted in higher TAV and CoPS outcomes. Nevertheless, large effect sizes were found for TAV, even for BoS variation. Moreover, the largest effect sizes for both perturbation amplitude and BoS width were found for TAV, indicating that TAV is a responsive outcome for balance assessment.

Whilst force plates offer detailed insights into overall balance performance, they do not provide insights in kinematics [17]. Therefore, clinical interpretation and translation to therapeutic goals is challenging. Interpretation of TAV is more straightforward, since it is the direct measurement of trunk movements. This facilitates clinicians’ understanding of the outcome and enables them to provide valuable feedback during training. Therefore, we suggest that the measurement of trunk movements during postural stability tests can provide added value beyond what is captured with force plate-based outcomes.

Some limitations of this study need to be addressed. The first limitation of this study is the relatively small sample size (N = 14). Despite this, we observed highly significant correlations between TAV and force plate-based outcomes (p < 0.001), as well as strong effects of perturbation magnitude on both TAV and force plate-based outcomes (p < 0.001), suggesting that statistical power was sufficient for these comparisons [27]. However, the power to detect effects of changes in base of support (BoS) width may have been limited, as significant effects were only observed for CoPS and TAV (p = 0.02 and p = 0.003, respectively). In this study, we manipulated perturbation magnitude and BoS width to modulate balance task difficulty and assess the responsiveness of TAV relative to force plate-based measures. Notably, the variation in perturbation magnitude induced a more substantial differentiation in balance difficulty compared to changes in BoS width. Moreover, the responsiveness of TAV was even better than the responsiveness of force plate-based outcomes. Importantly, the study was not designed to specifically detect and test for significant effects of perturbation amplitude and BoS width on dynamic postural stability. The second limitation of this study is the generalizability of the results, based on the specific population in this study. The mean age was quite low (21.9 years old) and the sport participation was high (4.3 times average a week). This may have influenced the results. The third limitation is the large imbalance rates for lateral perturbations. For that reason, only medial perturbations were included in our data analysis, in which the imbalance rates were much lower. This is consistent with the literature where it was shown that recovering from a lateral perturbation is more difficult than from a medial perturbation [28]. Imbalance rates of 46% and 10% were found for lateral and medial perturbations, respectively, in an earlier study [28], which closely aligns with the percentages of 49% and 10% in our study. A possible explanation for the large imbalance rates for lateral perturbations could be found in the differences in rotations in the frontal plane that occur due to perturbations in the medial and lateral directions. During a medial perturbation, the body rotates in the lateral direction, relative to the stance leg. For an effective stepping strategy, the swing leg should be placed laterally of the stance leg, which is practically possible, however not the most logical strategy. It is thought that, in the absence of an effective stepping strategy, participants are more prone to perform within the maximal capabilities of other balance strategies on one leg. During a lateral perturbation, the body rotates in the medial direction, causing the standing leg to adduct. The adduction movement of the standing legs brings the foot of the swinging leg closer to the ground, making it easy and effective to regain balance by touching the force plate with the swinging leg. It appears that participants chose a stepping strategy over an ankle- or hip strategy during lateral perturbations, although participants were asked to only place their swinging leg on the ground if necessary. Future research should take this into account. The fourth limitation concerns the range of manipulations of BoS width. An additional analysis of the maximum mediolateral amplitude of the CoP, using a repeated measures ANOVA, revealed no significant change when the BoS width was altered (p = 0.07). The average maximum mediolateral amplitude of the CoP was 5.1, 5.2 and 5.3 cm for the smallest (6 cm), medium (9 cm) and largest (12 cm) BoS width, respectively. The smallest BoS width in our study was 6 cm, which is wider than the maximum mediolateral amplitude of the CoP on all wooden footboards. Possibly, effects of altering the BoS width would have been larger if a BoS width smaller than 6 cm would have been added. The fifth limitation is the generalizability of our findings. Firstly, TAV has been found a convenient substitute for force plate-based postural stability outcomes. Whilst it is a simple and low-cost measure, our current dynamic postural stability test still required a platform perturbation device, which limits the immediate application of sensor-based postural stability testing. Secondly, it should be noted that we only investigated this relation during mediolateral platform perturbations in unipedal stance in this study. Further research is necessary to determine if the relation of several force plate-based outcomes and TAV is also present for balance challenges that are more related to sports conditions, such as landing from a drop jump.

## 5. Conclusions

Trunk angular velocity appears to be a promising outcome to use in the clinic. We observed moderate to strong correlations between force plate-based outcomes and trunk angular velocity after perturbations of the surface during single leg stance, supporting the concurrent validity of trunk angular velocity. Particularly the correlation between both center of pressure speed and the mean force variation of the vertical component of the ground reaction force and trunk angular velocity was strong. Larger perturbations and a reduced base of support both resulted in increased balance disturbances. The large effect sizes for trunk angular velocity in response to changes in perturbation magnitude and base of support width in the ANOVA suggests that trunk angular velocity is a responsive measure for balance assessment.

## Supporting information

S1 Fig Scatterplots of force plate-based postural stability outcomes and TAV of a typical participant. Each dot represents one medial perturbation, resulting in 72 dots per scatterplot. BoS widths of 12, 9 and 6 cm are represented in blue, orange and grey respectively.
(TIFF)
