# Supplementary figures and images for: Trunk angular velocity: A convenient, valid and responsive substitute for force plate-based measures of dynamic postural stability
Source: PLoS One. 2025 May 27;20(5):e0323993. doi: 10.1371/journal.pone.0323993 (PMC12111669; doi:10.1371/journal.pone.0323993)

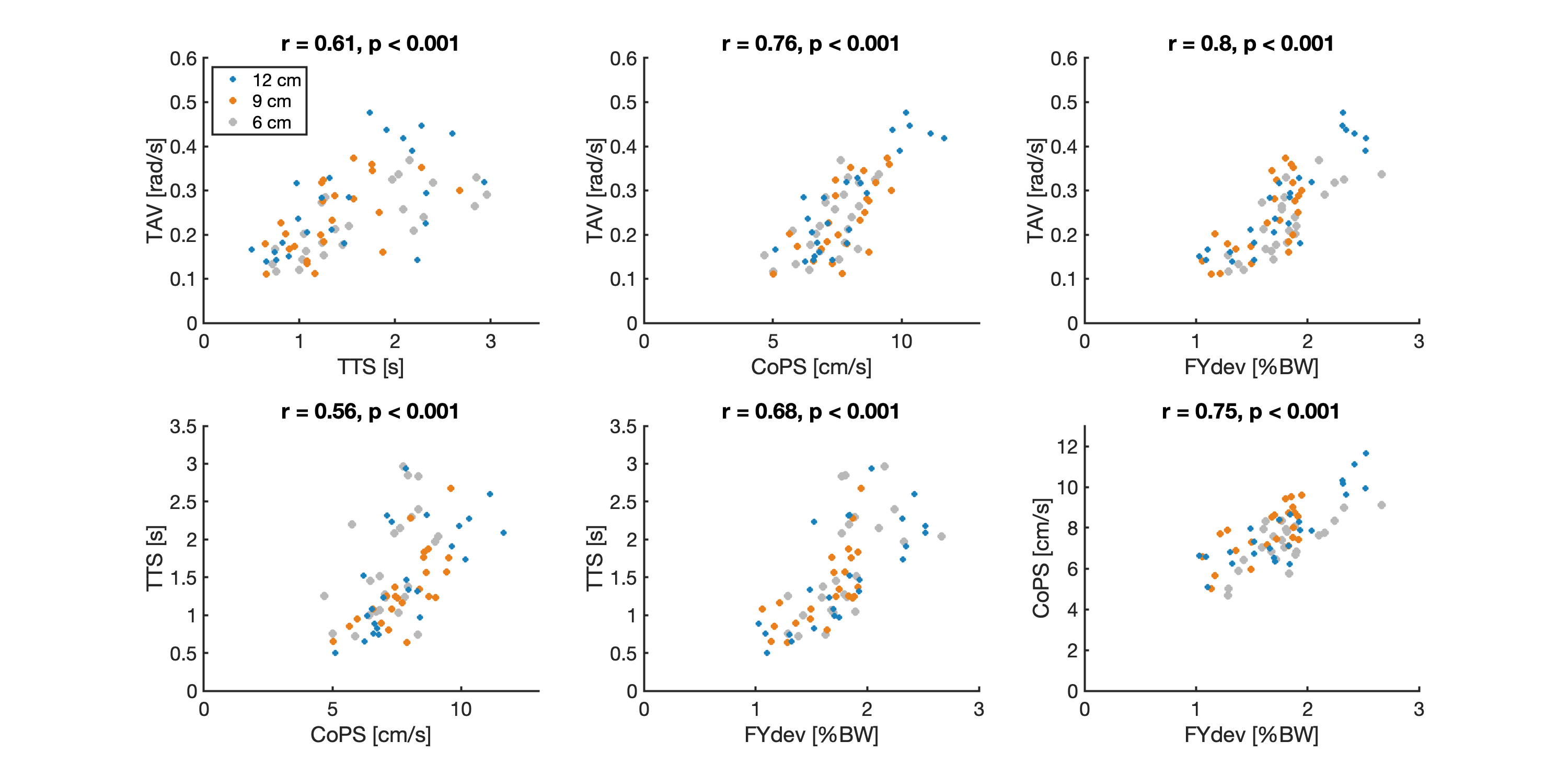

Supplement: S1 Fig — Each dot represents one medial perturbation, resulting in 72 dots per scatterplot. BoS widths of 12, 9 and 6 cm are represented in blue, orange and grey respectively. (TIFF) [file pone.0323993.s001.tiff]
